# Fibrillogenesis and Hydrogel Formation from Fibrinogen Induced by Calcium Salts

**DOI:** 10.3390/gels9030175

**Published:** 2023-02-22

**Authors:** Dominik Hense, Oliver I. Strube

**Affiliations:** Institute for Chemical Engineering, University of Innsbruck, Innrain 52c, 6020 Innsbruck, Austria

**Keywords:** fibrinogen nanofibers, fibrillogenesis, hydrogels, self-assembly, calcium, factor XIII, binding sites

## Abstract

Fibrin is considered a highly promising biomaterial for manifold medical applications. Although it is a well-established material in this field, the required enzyme thrombin bears some striking downsides such as high costs and health risks. Current research discovers more and more ways to use fibrin’s precursor fibrinogen as a substitute. Fibrinogen’s full potential is, however, only retained when using it as fibrous gel, as it is the case for fibrin. In our previous work, we introduced such a kind of material for the first time. This material, called pseudo-fibrin, shows striking similarities to fibrin regarding its supramolecular structure and is created in a facile salt-induced process, which we further improved in this study. In particular, we shine light on the role of Ca^2+^ in pseudo-fibrin buildup, which turned out to drastically improve the outcome. Never before has it been observed that Ca^2+^ can induce fibrillogenesis and the gelation of native, enzyme-free fibrinogen. Enzyme catalysis was ruled out by the addition of thrombin and factor XIII inhibitors. Even more striking, Ca^2+^ induces gelation even under physiological conditions, leading again to stable and fibrous hydrogels. Although this latter approach is possibly co-induced by residual factor XIII, the resulting gels are for the first time recognized as promising materials and not discounted as unwanted side effects. The finding that these gels again consist of fibers especially renders a new perspective on the role of factor XIII and fibrinogen’s well-known Ca^2+^ binding sites. In this study, we aim to provide first insights into this highly feasible material and its characteristics.

## 1. Introduction

The role of biobased and bioinspired materials in modern research expands continuously and covers increasingly broader areas of our everyday life. Decades ago, the potential of these materials was discovered, for example their use as casein-based paints [1]. More recent approaches provide a versatile range of applications, ranging from mussel- and gecko-inspired reversible glues [2] over gels [3,4] to the nanoscale structuring of surfaces via enzyme-mediated addressing of casein [5,6,7] or melanin [8,9,10].

One biomaterial of high interest is fibrin, a fibrous protein gel responsible for wound healing. Due to its function as a wound sealant, fibrin inherits a unique combination of properties, being especially non-toxicity and strongly adhesive [11]. These features are supplemented by a gel structure, resulting in a highly potent material for various applications. Fibrin is formed in an enzyme-catalyzed reaction, which involves its precursor fibrinogen as well as the enzyme thrombin. Briefly, thrombin cleaves two so-called fibrinopeptides (“A” and “B”), leading to a spontaneous polymerization of the fibrin monomers to protofibrils and eventually the three-dimensional fibrin networks [11,12]. Since both thrombin and fibrinogen are readily available for in vitro experiments, versatile research considers fibrin for medical applications. Among the topics of high interest are, for example, fibrin-based sealants in surgery [13,14], scaffolds in tissue-engineering [15], or substrates in bioprinting [16]. However, it became more and more clear that fibrin inherits some downsides, which arise mainly due to the required thrombin. Besides its high costs, there can be medical risks in the in vivo application of fibrin, especially the risk of thrombosis. Moreover, to prevent allergic reactions, the thrombin as well as the fibrinogen have to stem from humans [13,17]. This restriction lowers fibrin’s potential significantly. However, most of fibrin’s outstanding material properties stem from its precursor, therefore fibrinogen-based materials are considered as highly promising alternatives in current research.

Fibrinogen itself is a rod-like, 340 kDa large glycoprotein, which consists of two pairs of amino acid chains, labeled α, β, and γ. These chains are connected by disulfide bridges in the central region of the molecule. Additionally, the fibrinopeptides A and B are attached to chains α and β, respectively, which is why fibrinogen is also denoted as (AαBβγ)_2_ [11,12]. A further molecular feature is fibrinogen’s αC region, an extension of the α chains which is under physiological conditions non-covalently bound to the central domain. As a consequence of fibrinopeptide B cleavage, these αC regions unfold and promote lateral aggregation of the (proto)fibrils to gain thicker fibrin fibers [18].

In general, fibrinogen-based materials can be subdivided into several categories, depending on their gel-like or fibrous nature. Non-gelled and non-fibrous fibrinogen is mainly used in drug delivery [19,20,21] but also showed high potential when used as scaffolds for in vivo bone repair [22]. These kinds of materials focus on fibrinogen’s biocompatibility and disregard a lot of its full potential. Therefore, methods to induce fibrillogenesis of fibrinogen are intensively studied. Indeed, several approaches can be found to yield (enzyme-free) fibrinogen fibers, although not all of them are based on a self-assembly process like actual fibrin. A common way to create fibrinogen fibers is via electrospinning [23,24]. This process can be enhanced for example via the cross-linking of the proteins [25] or by using composite materials [26]. The created fibrinogen mats can be of high interest in tissue engineering [23].

A similar method yields fibrinogen fibers via an extrusion-like process. Again, highly concentrated fibrinogen solutions are used, which were channeled through a long, thin tube [27].

Many actual self-assembly processes to gain enzyme-free fibrinogen fibers require a substrate, i.e., surfaces on which fibrillogenesis takes place. In a recent work, fiber formation was observed upon drying when salts are present. This approach yields fibrous fibrinogen scaffolds [28,29]. Subsequent work by the same group showed that divalent metal cations do not trigger this process [30].

Similar to this approach, several studies reported a substrate-driven fiber formation, which however does not involve salts. Instead, the successful fibrillogenesis solely requires hydrophobic substrates such as polystyrene. This process is most probably mediated by fibrinogen’s αC region [31,32].

Enzyme-free fibrillogenesis in solution has been observed for decades but none of the methods lead to actual hydrogels. One early example of salt-induced fibrillogenesis was presented by Gollwitzer et al. in 1983, who dialyzed fibrinogen solutions against buffers with low ionic strength [33].

A more recent method to induce fibrillogenesis in solution is by adding ethanol to a low-concentrated fibrinogen solution [34,35]. Hydrogels however are not accessible with this method due to the low fibrinogen concentrations of 50 mg/L at most. Moreover, fibrinogen is denatured via this method, resulting in more amyloid-like fibers instead of retaining the natural conformation.

Recently, we introduced a new process to yield a fibrous, gel-like fibrinogen material in solution. This is the first method to gain a material close to fibrin in solution. The material called “pseudo-fibrin” is created by adding a defined amount of kosmotropic anions to a cold, salt-free fibrinogen solution at a slightly acidic-to-neutral pH. Pseudo-fibrin can also be lyophilized to gain highly porous aerogels, therefore filling the gap in search for a convincing fibrin alternative [36].

To further analyze this fascinating material and its underlying mechanism, we now characterize the impact of Ca^2+^ as it already plays an important role in the blood clotting cascade. It is known that fibrinogen has some binding sites for these ions, and the binding of Ca^2+^ is especially known for significant material improvements. These improvements include stabilization against acid and heat denaturation [37] and stabilization against plasmin degradation [37]. Ca^2+^ ions even accelerate fibrin formation [11,38].

In the 1980s, Steven et al. had already reported a gelation of fibrinogen in the presence of some divalent metal cations, for example Fe^2+^, Cu^2+^, and Hg^2+^. Interestingly, the authors emphasize that Mg^2+^ and Ca^2+^ especially do not induce any kind of gelation under the applied conditions [39]. There are however some rather unusual side-effects of Ca^2+^ reported in the literature. We rediscovered a 40-year-old approach to trigger an (apparently) Ca^2+^-induced gelation of fibrinogen. Rapidly, this phenomenon could be explained by the residuals of the enzyme factor XIII in the purchased fibrinogen. Factor XIII, i.e., its activated form factor XIIIa, is normally responsible for the covalent cross-linking of fibrin [40]. Years later, the factor XIII-induced gelation of fibrinogen was further investigated by another working group, who were interested in the question: How exactly could Ca^2+^ activate factor XIII, since this process usually requires thrombin? This is also the natural pathway; thrombin and Ca^2+^ activate factor XIII to form factor XIIIa. The researchers found out that an activation of the enzyme is indeed possible solely via Ca^2+^, but requires concentrations of at least 50 mmol/L [41,42]. In all cases, nothing was reported regarding the microscopic structure of the gels. Additionally, the potential of this gel as an actual material was disregarded.

Another interesting finding regarding Ca^2+^ was reported by Marx, who found that fibrin protofibrils (not the monomers) form a fibrous clot when exposed to 1–2 mmol/L Ca^2+^. This process crucially requires fibrin protofibrils which have to be created beforehand via thrombin addition and nearly instant quenching. Only in this case is Ca^2+^ able to “continue” self-assembly. Zn^2+^ ions have an even more pronounced effect. This procedure does not involve any factor XIII, solely thrombin (in the beginning) and Ca^2+^ [43,44]. Later, Marx found that the cleavage of fibrinopeptides A and B exposes additional binding sites for Ca^2+^ and Zn^2+^, respectively. Consequently, it is not the presence of protofibrils that is crucial for the process; the cleavage of fibrinopeptide A simply inevitably leads to such an outcome [45].

All these insights into Ca^2+^-related effects show how much of an influence it has in many aspects of blood clotting. Although it inherits such enormous potential, it has never been observed before that Ca^2+^ itself can trigger fibrillogenesis and gelation at the same time. This effect is most likely linked to the occupancy of binding sites under suited reaction conditions and potentially renders a new perspective on their role and functions. Further, this observation potentially fills the gap between all reported Ca^2+^-related self-assembly approaches.

We herein aim to introduce a new approach to create fibrous fibrinogen hydrogels, which emerge by adding Ca^2+^ to the protein solution. To get an impression of this material, gels are analyzed mainly via scanning electron microscopy. First insights into the mechanical properties are provided by measuring the rheological behavior.

## 2. Results and Discussion

### 2.1. Earth Alkaline Metal Chlorides

Our previously introduced anion-driven process to create fibrin-like pseudo-fibrin gels requires kosmotropic anions. We now aim to characterize the impact of divalent cations on this approach, as they play an important role in the natural blood clotting process. To exclude anion effects, first the experiments were performed using divalent metal chlorides such as MgCl_2_, CaCl_2,_ SrCl_2_, and BaCl_2_. The reaction time was expanded to 24 h to not oversee any effects, otherwise all conditions were kept identical to the standard “anion process” (5 °C, 15 mmol/L of the trigger salt, no other salts/additions, pH 7.0). As already known from previous studies, a white precipitate is formed upon salt addition. By stirring, these (and all potentially emerging fibers) aggregate and settle down to the bottom, so this has to be strictly avoided [36].

**Figure 1:**
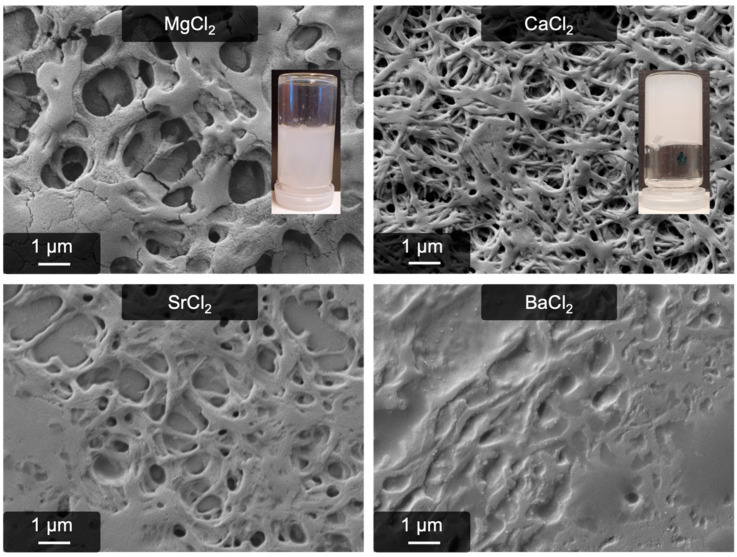
Comparison of pseudo-fibrin created with MgCl_2_, CaCl_2_, SrCl_2_, and BaCl_2_. Only CaCl_2_ yields high amounts of fibers; in all other cases, unstructured precipitates dominate. Further, these gels are not as stable as the calcium one. Inserted are photographs of the Ca^2+^-induced gel and the Mg^2+^-induced gel (representative for all other earth alkaline metals).

Surprisingly, in all cases a gel-like material was formed after 24 h, which was significantly more stable than the ones gained in all our previous experiments. In comparison to fibrin, these are completely white and nontransparent, whereas fibrin under the same conditions shows only slight turbidity. This observation is already a hint that a new material was created here. The gels with CaCl_2_ as the trigger are remarkably stable especially, whereas the others appeared to be simply precipitated. Scanning electron microscopy of the four samples revealed a fibrous structure in the case of CaCl_2_, as Figure 1 shows. Samples with MgCl_2_, SrCl_2_, and BaCl_2_ were less defined and contained more unstructured fibrinogen. These findings were not described before; on the contrary, Mg^2+^ and Ca^2+^ were explicitly mentioned to not induce gelation or fibrillogenesis of fibrinogen [39].

Ca^2+^-induced gelation increases the potential of pseudo-fibrin even more, since actual gels are now easily accessible with non-toxic salts. It is, however, unclear whether this material is actually the same as our previously described pseudo-fibrin. To take a deeper insight into this issue, further experiments with calcium salts were performed.

### 2.2. Calcium-Induced Gelation

Replacing the sodium phosphate buffer of our anion-triggered fibrillogenesis with the same amount of CaCl_2_ yields a fibrin-like hydrogel with a remarkably fibrous sub-structure. This finding brought up some fundamental questions regarding the role of Ca^2+^ in this process. The first important step is to investigate whether the process involves unintended enzyme catalysis. It is known that the activation of several blood-clotting factors requires calcium [12,46]. To rule out accidental activation of such potential residuals, the supplied fibrinogen was analyzed via SDS-PAGE, but no contaminants were found (Figure S1). To be certain about this, Ca^2+^-induced self-assembly was repeated in the presence of the thrombin inhibitor AEBSF and factor XIII inhibitor 2-iodoacetamide. Both experiments still lead to the same result. The latter especially proves that our Ca^2+^-induced approach is different from previously described ones, which either use protofibrils [45] or factor XIII [40].

Although enzyme catalysis can be ruled out as a trigger, it is not clear at this point what specifically leads to the self-assembly of fibrinogen to fibers. Most probably, this process is solely induced via Ca^2+^ ions since neither NaCl nor other earth alkaline chlorides lead to such results. Indeed, a similar experiment performed with Ca(NO_3_)_2_ leads to the same fibrous gel, strengthening the hypothesis of a calcium-induced process. Most calcium salts, however, are not soluble to such a high extent, which hinders comparison with various calcium salts. A combination with kosmotropic anions would be interesting to fill the gap between the already introduced “anion process” and the calcium one.

**Figure 2:**
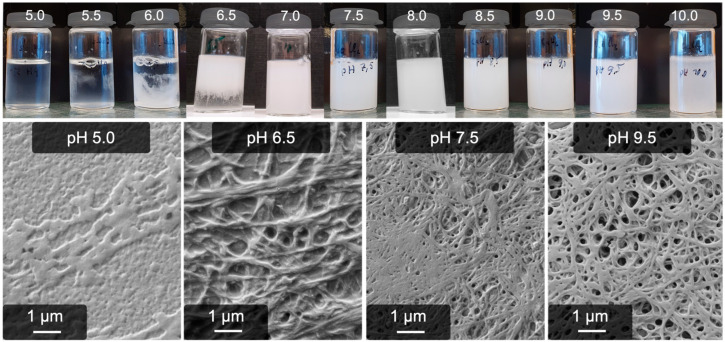
Influence of pH on calcium-induced gelation. At a pH < 6.5, no gelation occurs; at a pH of 6.5, the gelation is incomplete, and only at a pH of 10 does the gel begin to become instable again.

The similarities and differences to this anion process were studied in detail by means of the pH dependence, time dependence, ion concentrations, and temperature. The high amount of fibers and the improved gel stability already allow the assumption that the calcium process may be more robust than the anion one. Indeed, as Figure 2 shows, the process works well at an alkaline pH; even at a pH of 9.5, a stable gel and high amounts of fibers are obtained (the solubility of Ca(OH)_2_ is around 23 mmol/L, which is not exceeded). On the other hand, the gelation becomes rapidly incomplete below a pH of 7.0. Also, the resulting fibers become unstructured until the process is inhibited at a pH of 6.0. SEM images of all further samples are provided in the supporting information (Figures S2 and S3).

**Figure 3:**
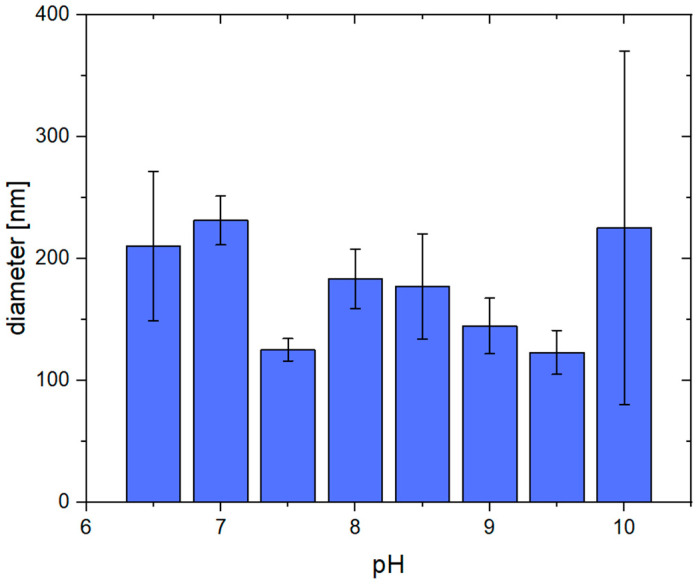
Average diameters of pseudo-fibrin fibers at different pH. At pH < 6.5, no fibrous structures are obtained anymore. At a pH of 10, the fibers become less defined, resulting in a broad range of diameters.

In addition to the SEM images, the average fiber diameter was determined at each pH. As can be seen in Figure 3, the average diameters are very similar and mostly vary around 150 nm. At a pH of 10, only a few fibrous structures can be recognized, which, however, are larger than the ones at a lower pH. By trend, if the average diameter exceeds 200 nm, the structures are less fibrous (pH 6.5, 7.0, and 10.0). The diameters, including their standard deviation, can also be found in the supporting information (Table S1).

Although the mechanistic details have not been revealed yet, a reasonable explanation for this phenomenon lies in the occupancy of Ca^2+^ binding sites, which mostly vanish at an acidic pH due to protonation [47].

**Figure 4:**
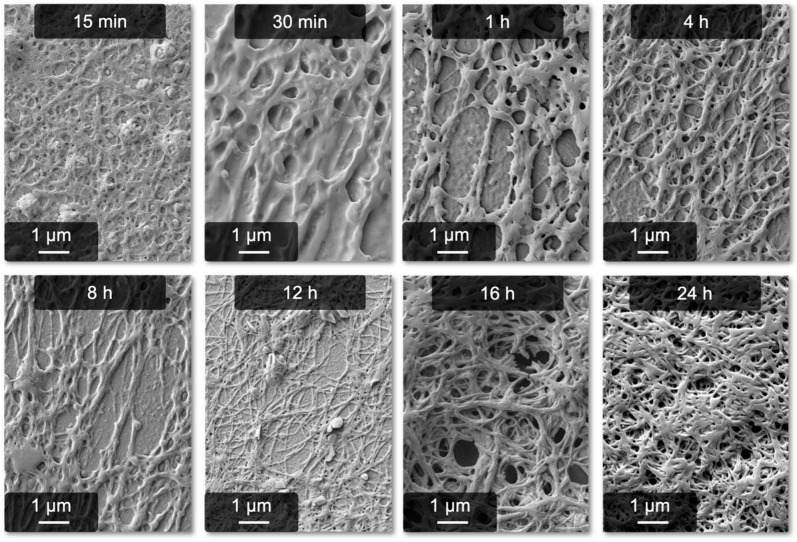
Progress of the Ca^2+^-induced fiber formation from 15 min after CaCl_2_ addition to 24 h.

When preparing the samples, it can immediately be noticed that the reaction time is extended compared to the anion process. While the latter is complete after approximately 4 h, the calcium-induced gelation takes significantly longer. To gain more insight into the buildup of the fibers, a time series from 15 min to 24 h was performed. The results, displayed in Figure 4, clearly confirm the expanded reaction time. Interestingly, it takes nearly 4 h to build up the first defined fibers, although the first fibrous structures can be observed after 30 min. After around 16 h, the process can be considered complete. Besides this finding, the time series proves that the fibrillogenesis actually happens in solution and does not require further drying steps or substrates. Otherwise, all samples would look identical since the chemical composition does not vary between the samples. Further insights into this issue were already provided in our previous work, in which we compared lyophilized pseudo-fibrin with fibrin [36]. Both materials show a similar structure in the SEM, leading to the conclusion that fibrillogenesis is not related to drying or surface-induced effects.

**Figure 5:**
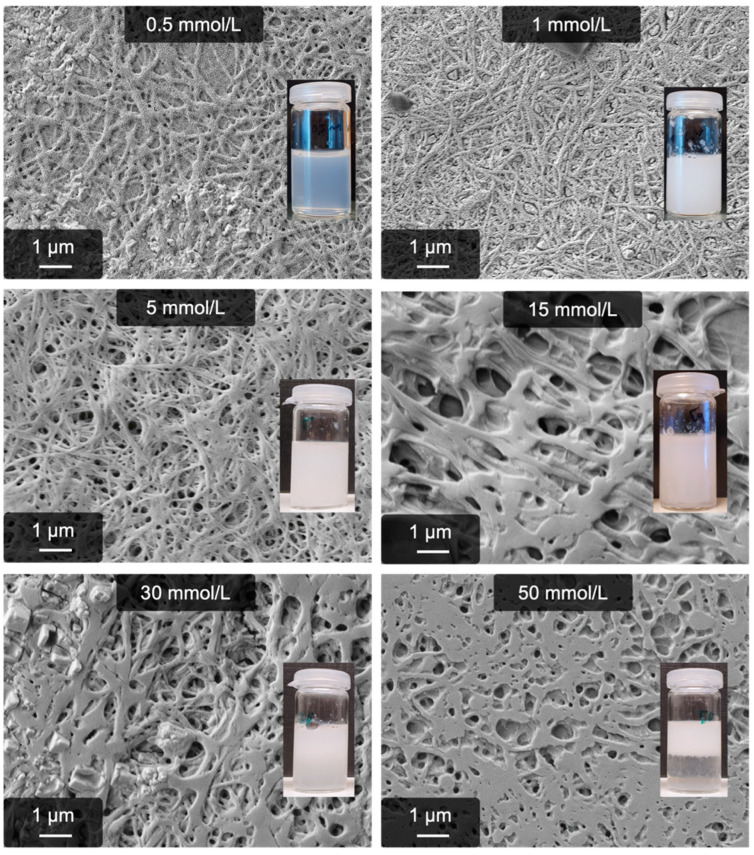
Pseudo-fibrin created with different CaCl_2_ concentrations. Even with 1 mmol/L, high amounts of fibers are created.

As a next step to characterize the calcium process, the optimal CaCl_2_ concentration is determined. The calcium process has already proven to be very resistant against alkaline pH, so an increased stability at a high ion concentration can be expected. The results were even more surprising, especially regarding the lower end of ion concentrations; even with 0.5 mmol/L, significant amounts of fibers (but no gel anymore) are obtained. A gel is, however, still formed with 1 mmol/L Ca^2+^. The upper limit is around 30 mmol/L Ca^2+^ and is therefore increased compared to the anion process. The corresponding microscopy images are presented in Figure 5.

**Figure 6:**
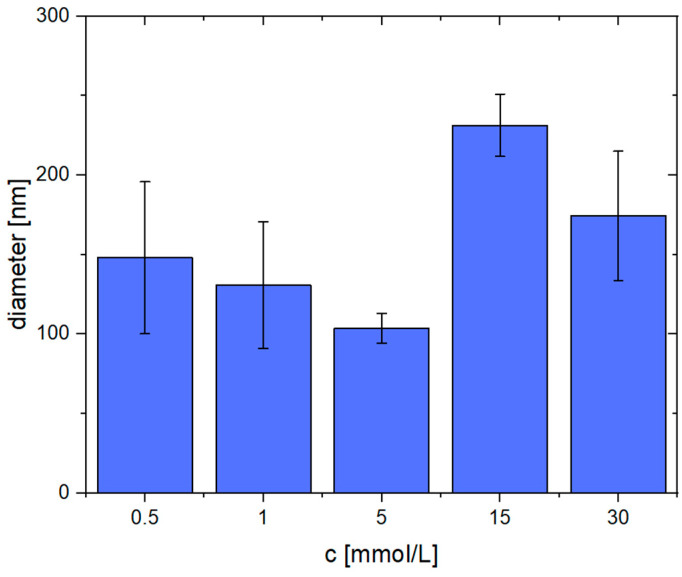
Average diameters of pseudo-fibrin fibers at different Ca^2+^ concentrations.

Again, the average fiber diameters were determined. These are shown in Figure 6. When using low Ca^2+^ concentrations (0.5–5 mmol/L), the diameter is in the range of 100–150 nm, whereas the sample created with 5 mmol/L Ca^2+^ shows the lowest standard deviation (104 ± 9 nm). Beginning at 15 mmol/L Ca^2+^, the obtained fibers appear more coalesced and are thicker, i.e., the diameter is approximately 200 nm. The average diameters and their standard deviation can also be found in the supporting information (Table S2).

Another way to achieve high ion concentrations is by adding further salts, most commonly NaCl. This is mostly done to mimic physiological conditions (140 mmol/L NaCl) and to increase the solubility of fibrinogen. The latter was analyzed in detail by means of time-resolved light scattering in our early works regarding fibrinogen self-assembly [48]. Briefly, it was shown that high amounts of NaCl slow down or inhibit salt-induced self-assembly processes in solution. However, the calcium process even works in the presence of high amounts of NaCl; it is simply slowed down. Gelation can be observed even at 140 mmol/L after several days of reaction time, but the resulting product is rather unstable.

It is not surprising that also elevated temperatures (37 °C) do not inhibit the process either, but significantly decrease yield and kinetics. The product is however less stable and can hardly be considered as “gel-like”. On the other hand, pseudo-fibrin produced at 5 °C only partially redissolves at 37 °C. Regarding the weakness against high ion concentrations and temperature, it becomes clear again that the process cannot be enzyme-driven, especially since the physiological temperature would rather enhance enzyme activity. Furthermore, this brings up a new explanation as to why no Ca^2+^-triggered fibrillogenesis has been reported yet; the combination of the high salt concentration, physiological temperature, and rather low Ca^2+^ concentrations disrupts the process so much that it was overlooked. Finally, many studies regarding fibrin are conducted in phosphate buffered saline, which is incompatible with such high amounts of Ca^2+^. All these aspects together are the reason why this phenomenon has not been described before.

It is highly likely that this process is linked to the well-known binding sites for Ca^2+^; at least, it is very probable that the Ca^2+^ ions occupy them. In general, the specific and unspecific binding sites must be distinguished. The unspecific ones are electrostatic effects due to the net charge of fibrinogen at alkaline pH. These ones are occupied by Mg^2+^ as well as Ca^2+^, which is why they are labeled unspecific. The specific ones, however, are only suited for Ca^2+^ as they involve chelate complexes [47]. As a result, the molecule conformation changes [49], leading to interesting side effects such as enhanced stability against denaturation and degradation [37]. The significant difference between Mg^2+^- induced gels and Ca^2+^-induced gels is most likely attributed to the occupancy of these high-affinity binding sites, which renders a fascinating new perspective on their role and function in general. Although the exact mechanism(s) of gelation and fibrillogenesis are not clear yet and require deeper investigation (for example via light scattering and IR measurements), we hypothesize the following contribution: theoretical work regarding the charge distribution of fibrinogen show that the outer domains of the protein (the D domains) are negatively charged under physiological conditions [50]. Consequently, divalent cations could interact with the protein at these outer positions and potentially favor aggregation via the “bridging” of two neighboring molecules. High ion concentrations would, however, favor the (monomeric) dissolution of the molecules due to the increased ionic strength. This however cannot be the complete mechanism of pseudo-fibrin formation, because otherwise fibrillogenesis and gelation would be independent of the respective (divalent) cation. As seen previously, this is not the case, as Ca^2+^ triggers a unique effect. Most likely, fibrinogen undergoes changes in its secondary structure upon Ca^2+^ addition, which already has been observed in several previously mentioned studies. These changes potentially favor a more orientated aggregation, in this case to fibers. Other ions such as Mg^2+^ or Sr^2+^ presumably do not fit into the molecular structure because the specific binding sites are intended for Ca^2+^, i.e., cations of this respective size. These aspects however have to be proven in future work.

In summary, the Ca^2+^-induced fibrillogenesis proved to be a facile and even more convincing way to create a fibrin-like material without enzymes. The reaction conditions are more flexible compared to our anion process and include an extended regime of ion concentrations, reaction temperatures, and pH. Only the reaction time is increased to at least 16 h. Most important, it could be shown that this process is neither enzyme-induced nor related to substrates or drying effects.

### 2.3. Calcium-Induced Gelation under Physiological Conditions

The Ca^2+^-induced pseudo-fibrin is significantly more robust against ion concentrations and temperatures than our previously introduced anion process. However, a limitation until now is the necessity for low temperatures and salt concentrations for optimal yield and kinetics. Ideally, the gels can even be fabricated under physiological conditions. Since the process is in principle possible at 37 °C (but with low yield and disadvantageous kinetics), the first step to counterbalance these effects is a much higher concentration of fibrinogen (and consequently of product). We therefore performed a new series of experiments to finally achieve gelation at 37 °C.

The overall robustness of the Ca^2+^ system allows for the use of a buffered system to stabilize the pH at 7.5. For this purpose, 4-(2-hydroxyethyl)-1-piperazineethanesulfonic acid (“HEPES”) was chosen. To not disrupt the process too much initially, experiments were carried out without additional NaCl at 37 °C. Dissolution of 20 g/L fibrinogen— fourfold of the previously possible concentration—was achieved rather quickly (approximately 10 min). The addition of the previously successful 15 mmol/L CaCl_2_, however, only leads to a slight turbidity, followed by unspecific precipitation. This precipitate settles down to the bottom of the reaction vessel. Even after 24 h of reaction time, no gel or fibers are formed.

However, increasing the Ca^2+^ concentration to 25 mmol/L Ca^2+^ changes the outcome, as a genuine gel formation is observed. Even better results are obtained when using 50 mmol/L Ca^2+^: After 24 h, a stable gel has emerged. Still, even when using this high Ca^2+^ concentration, an initial precipitate forms directly upon salt addition. Although the samples do gel, the precipitation is undesirable because less fibrinogen is available, and the overall control is lower. To prevent this, 100 mmol/L NaCl are added, which also brings the system even closer to physiological conditions. As a side effect, the high amount of NaCl allows us to dissolve even more fibrinogen. Indeed, 40 g/L of fibrinogen can be brought into solution within minutes using this approach.

**Figure 7:**
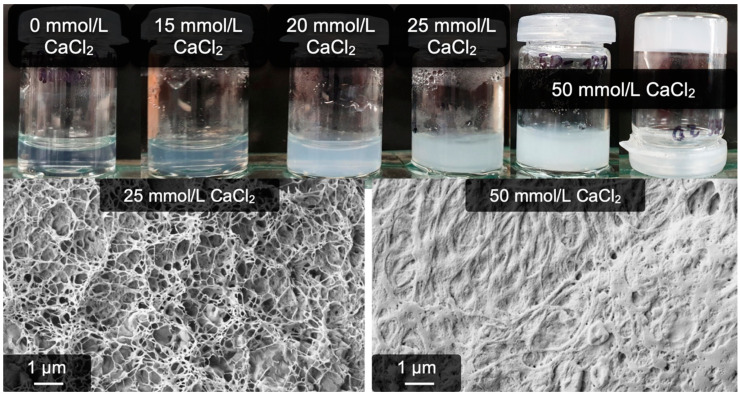
Calcium-induced fibrinogen hydrogels after 24-h reaction time. From left to right, the CaCl_2_ concentrations are 0 mmol/L, 15 mmol/L, 20 mmol/L, 25 mmol/L, and 50 mmol/L. Stable gels are obtained with a minimum of 25 mmol/L CaCl_2_. All samples contain additionally 100 mmol/L NaCl.

Figure 7 shows the results of a CaCl_2_ concentration series with additional NaCl. It can clearly be seen that 15 mmol/L of CaCl_2_ is again not sufficient to induce gelation. The first stable gels are obtained with 25 mmol/L of CaCl_2_. A further increase to 50 mmol/L of CaCl_2_ is accompanied by a more complete gelation, as can already be determined by eye regarding the intense white appearance of the gel. The reference sample without calcium did not undergo any change and remained transparent. The microscopic structure of these gels is also shown in Figure 7 and again reveals fibrous structures of the gels. At 25 mmol/L of Ca^2+^, filigree networks are seen, and using 50 mmol/L of Ca^2+^ yields high amounts of fibers. These appear more undefined, which is however just a side effect of the high fibrinogen concentration combined with the drying step necessary for electron microscopy. An analogously prepared reference sample of fibrin yielded similar results, i.e., many fibers were incorporated into a rather dense clot (see Figure S4).

Minor adjustments of the process include pH adjustments and identification of the actually necessary reaction time. As already observed previously, a pH < 6.5 completely inhibits the process. Regarding the reaction time, the process is also analogous to the previously described one in Section 2.2, i.e., it can be qualitatively considered complete already after 16 h.

The question arose, Why is the gelation of fibrinogen so fruitful even under physiological conditions? Successful gelation requires significantly more Ca^2+^, as was the case for the previous gels at lower temperatures. This observation speaks for a contribution of fibrinogen’s well-known Ca^2+^ binding sites because more fibrinogen consequently binds more Ca^2+^ [47]. Another explanation are the potential residuals of the fibrin-stabilizing factor XIII remaining in commercially available fibrinogen. Such gelation was already observed in the 1980s and treated as an undesired side effect [40]. Indeed, the activation of residual factor XIII is possible with at least 50 mmol/L of Ca^2+^, although this does not resemble the natural pathway [42,51]. Our following control experiments with the factor XIII inhibitor iodoacetamide disrupted gelation and solely resulted in precipitation upon Ca^2+^ addition. This indicates that the described gelation under physiological conditions can be at least partially ascribed to factor XIII residuals, although some open questions remain. First, gelation already succeeds when using only 25 mmol/L of Ca^2+^—half of the required amount to activate factor XIII [51]. Even more striking, factor XIII-induced gels are explicitly described as non-fibrin-like regarding their three-dimensional structure [42]. Instead of a purely factor XIII-induced gelation, the observed phenomena at least interfere with the earlier introduced Ca^2+^-induced process at a low temperature.

Independent from the mechanism(s), however, the presented experiments provide an effective approach to gel commercial fibrinogen via Ca^2+^ addition. Residuals of factor XIII might even covalently cross-link the resulting fibers, which could explain its high stability and render this material a promising alternative to fibrin. This approach was for the first time considered as an efficient way to obtain a fascinating new material instead of an undesired side reaction.

### 2.4. Yield, Water Content, and Rheological Properties

After introducing this new approach to obtain calcium-induced hydrogels, the last step is to provide the first insights into their properties as a material.

**Figure 8:**
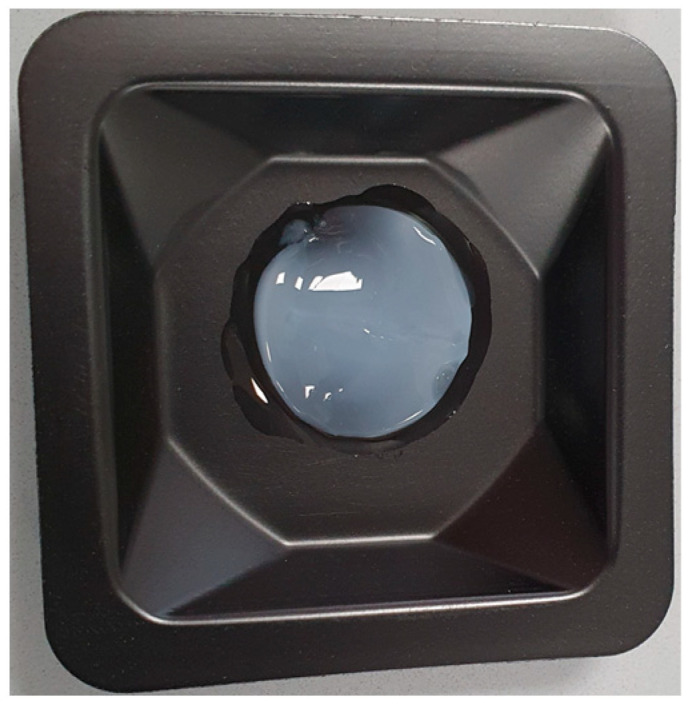
A Ca^2+^-induced fibrinogen hydrogel extracted from its original vessel. The gel could easily be extracted without damage.

First, the water content, yield, and purity of the pseudo-fibrin gels were determined. This requires careful extraction from the original vessel, which was easily possible without damaging the material. A photograph of the gel is shown in Figure 8. To get a better impression of the hydrogel, an additional video can be found in the supporting information.

To determine the water content, yield, and purity, the gels were either dried directly after their fabrication or after one additional day of storage in a large volume of fresh buffer. As can be seen in Table 1, the unwashed pseudo-fibrin still contains significant amounts of unreacted fibrinogen (i.e., 7.3 mg or 42%), which diffuses out during washing. The actual gel has a yield of approximately 50% and contains 46.2 mg of water per mg protein (corresponding to a water content of 97.9%).

**Figure 9:**
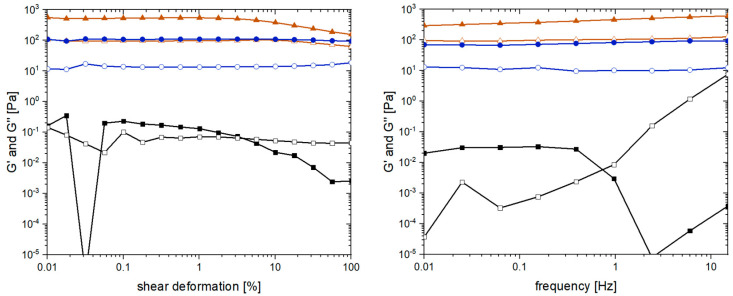
Rheological characterization of calcium-induced pseudo-fibrin (●) compared with non-cross-linked fibrin (▲) and native fibrinogen (■). Shown are the storage modulus G’ (closed symbols) and the loss modulus G’’ (open symbols).

As a last step to obtain the first insights into the “optimized” pseudo-fibrin gels, rheological measurements were performed. These measurements are a common strategy to monitor and compare various important characteristics of hydrogels such as mechanical stability, the degree of cross-linking, and reversibility of cross-linking [52]. The most relevant quantities are in this case the storage modulus G’ and the loss modulus G’’. As reference to pseudo-fibrin, non-cross-linked fibrin was chosen. The second reference is a solution of native fibrinogen to demonstrate the impact of Ca^2+^ addition. The results of the amplitude and frequency scan are shown in Figure 9.

The main purpose of the amplitude scan is to identify the linear viscoelastic regime of a material. For fibrin, G’ and G’’ are independent of the shear deformation until it reaches values of approximately 5%. Even macroscopically, this effect is very prominent as the water is pressed out of fibrin, leaving behind a dense protein clot. This effect is not observed for pseudo-fibrin, which shows constant storage and loss modulus. Regarding the absolute values, G’ and G’’ of fibrin are approximately six times higher than the ones of pseudo-fibrin. More precisely, G’ of fibrin is 600 Pa, whereas G’ of pseudo-fibrin is only 100 Pa. Before pseudo-fibrin can be considered as an actual alternative to fibrin, this issue has to be addressed in future studies. A potential strategy would be a subsequent covalent cross-linking of the gels. Remarkably, G’ and G’’ of pseudo-fibrin are far beyond the ones of native fibrinogen, demonstrating the difference Ca^2+^ addition makes; instead of a barely measurable solution (G’ is approximately 0.2 Pa), a stable gel is obtained.

When characterizing hydrogels via their rheological properties, the most common measurement is the frequency dependence of G’ and G’’. Again, fibrin’s storage and loss modulus still surpass the ones of pseudo-fibrin, indicating a higher degree of cross-linking. Since G’ and G’’ are nearly constant in both cases, the materials remain stable in the measured frequency range. In contrast, the solution of native fibrinogen is again only barely measurable (G’ = 0.03 Pa). Further, the curves of G’ and G’’ intersect at 0.7 Hz, which is not the case for fibrin and pseudo-fibrin.

The presented rheological measurements serve as a first insight into pseudo-fibrin’s material properties. Certainly, there are numerous further possibilities to adequately characterize the material, which go beyond the scope of this first work. We herein aim to introduce a new material and provide first insights into its structure. Deeper insights, including into the mechanism of gelation and the actual potential for (medical) applications, will be the subjects of future studies.

## 3. Conclusions

Exchanging the cation in our previously introduced process to gain enzyme-free “pseudo-fibrin” has a huge impact on the outcome. Starting with the addition of earth alkaline chlorides, it quickly became obvious that Ca^2+^ has a unique ability to induce an intense fibrillogenesis and gelation on its own, which has not been observed anywhere before. These hydrogels are remarkably stable and, most importantly, resemble the fibrous structure of actual fibrin. Unwanted enzyme catalysis, especially via thrombin and factor XIII, was ruled out as trigger, proving a purely salt-induced process. The calcium-induced process is more resistant towards temperature, ion concentration(s), and alkaline pH especially, than our previously introduced anion-driven process. The hypothesis arose that this “Ca^2+^ process” is based on the occupancy of the already known binding sites for divalent cations. Evidence for this hypothesis especially includes an inhibition of the process at an acidic pH, where most binding sites vanish.

Ca^2+^-induced fibrillogenesis is even possible under physiological conditions, resulting again in stable hydrogels. Although residuals of factor XIII in commercially available fibrinogen possibly contribute to this discovery, it still yields fibrous structures, opening up a new perspective on factor XIII cross-linking and the role of Ca^2+^. The hypothesis arose that this process still interferes with the previously described Ca^2+^-induced fibrillogenesis. Independent of the mechanism(s), these feasible and easy to produce gels were for the first time considered as actual materials instead of side effects.

To characterize pseudo-fibrin’s potential as an actual alternative to fibrin, profound further studies are necessary. These studies should especially include a detailed mechanical analysis, cell viability tests, and mechanistic insights such as possible changes in protein folding via IR measurements. In general, the findings open up a large new field of research, not only for material optimization but also for fundamental research regarding fibrinogen itself. The newly discovered influence of Ca^2+^ may even shine new light on the role of fibrinogen’s binding sites for divalent cations and contribute to an even more comprehensive view of the protein.

## 4. Materials and Methods

### 4.1. Materials

Fibrinogen from bovine plasma (≥95% protein of which ≥95% is clottable) was purchased from Merck. The protein was not further purified before use. Thrombin (50 U/mg solid) was obtained from Fisher scientific. 4-(2-hydroxyethyl)-1-piperazineethanesulfonic acid (“HEPES”, purity > 99.5%) was purchased from Carl Roth. 4-(2-aminoethyl) benzenesulfonyl fluoride hydrochloride (AEBSF; purity ≥ 98%) was bought from Carl Roth. 2-iodoacetamide (purity 98%) was obtained from thermo scientific. All experiments were performed in pure water (HPLC grade, specific conductivity max. 1 μS/cm) by VWR. All other chemicals were of at least 98% purity and purchased from the usual suppliers.

To prepare the salt stock solutions, the respective amount of salt was first weighed in and then dissolved in water. If necessary, the pH was adjusted to 7.0 using NaOH or HCl. If not stated differently, all salt stock solutions had a concentration of 500 mmol/L.

### 4.2. Production of Mg^2+^-, Ca^2+^-,Sr^2+^-, and Ba^2+^-Induced Hydrogels

Fibrinogen solutions with a concentration of 5 g/L were prepared by suspending 20 mg of the protein in 4 mL of 5 °C cold pure water. The pH was adjusted to 7.0 using 0.1 mol/L NaOH. The solution was stirred at 300 rpm for 10 min until all fibrinogen was dissolved.

Fibrinogen self-assembly was triggered by adding 120 μL of the respective 500 mmol/L of salt stock solution. Addition of the salt stock solution has to be performed carefully, i.e., fast but without stirring. This results in a salt concentration of 15 mmol/L, which served as reference in all experiments. For experiments using different salt concentrations, the volume of the added salt solution was adjusted accordingly. Finally, the reaction mixture was stored for 24 h at 5 °C if not stated differently. A 12 × 12 mm^2^ microscopy cover slide was used to collect some of the material for scanning electron microscopy. These samples were dried overnight at room temperature and ambient pressure.

Experiments using different pH were performed analogously; the required amount of NaOH/HCl to reach the desired pH was however added together with the CaCl_2_ solution. The initial fibrinogen solution was still prepared at pH 7.0.

For experiments using different CaCl_2_ concentrations, the added volume of the stock solution was adjusted accordingly.

**Figure 10:**
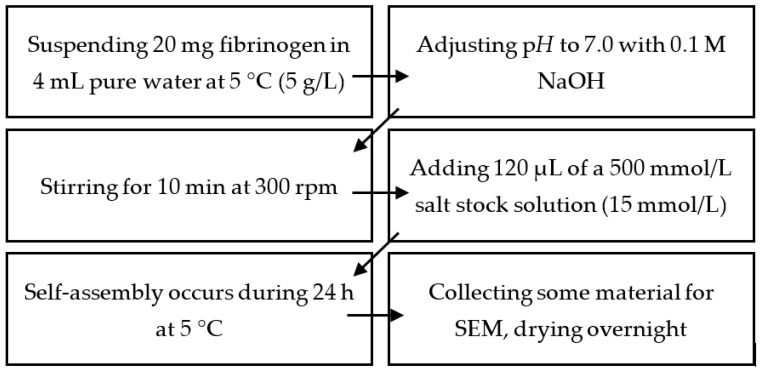
Summary of the necessary steps to prepare pseudo-fibrin. Experiments using a different pH than 7.0 were performed by mixing the respectively required amount of HCl or NaOH with the salt stock solution prior to its addition. Different salt concentrations were achieved by adjusting the volume of the added salt stock solution.

A summary of these steps is provided below in Figure 10.

### 4.3. Production of Fibrinogen Hydrogels under Physiological Conditions

A 50 mmol/L HEPES buffer stock solution was prepared by weighing in the required amount of solid, dissolving it in pure water and adjusting the pH to 7.5 with NaOH at 37 °C.

Preparation of pseudo-fibrin follows similar steps as described in Figure 10. However, the following changes have to be applied, mainly including changes in temperature and ion concentrations: 20 mg/mL of fibrinogen were dissolved in 20 mmol/L of HEPES buffer at a pH of 7.5. If an experiment was conducted in the presence of NaCl, the required 100 mmol/L of sodium chloride were already added to this precursor solution. The mixture was brought to 37 °C in a water bath. The fibrinogen dissolved completely during 10 min. As a last step, the respective amount of 500 mmol/L of CaCl_2_ solution was added to reach the desired Ca^2+^ concentration (typically 50 mmol/L). If different concentrations were used, the added volume was adjusted accordingly. Reaction mixtures were allowed to react for 24 h at 37 °C. The total reaction volume was in all cases 1 mL. A fibrin reference was prepared analogously but the CaCl_2_ was replaced by 5 U/mL thrombin.

After the respective reaction time, a 12 × 12 mm^2^ microscopy cover slide was used to collect some of the material for scanning electron microscopy. These samples were dried overnight at room temperature and ambient pressure.

### 4.4. Inhibition of Thrombin and Factor XIII

Thrombin catalysis was ruled out by adding 0.5 mmol/L of AEBSF to the reaction mixture prior to the respective trigger salt. Fresh stock solutions of 2-iodoacetamide in ultrapure water with a concentration of 1 mmol/L were prepared prior to each respective experiment. Factor XIII catalysis was ruled out by adding 0.1 mmol/L 2-iodoacetamide to the reaction mixture prior to the respective trigger salt. Both inhibitors were incubated for 10 min at 37 °C. Reference experiments, to control whether this reaction time is sufficient, lasted overnight at 37 °C but yielded the same results.

### 4.5. Fibrinogen and Water Content of Calcium-Induced Gels Produced under Physiological Conditions

Four pseudo-fibrin gels were prepared as described above (1 mL of gel containing 20 mg/mL of protein, 20 mmol/L of HEPES pH 7.5, 100 mmol/L of NaCl, 50 mmol/L of CaCl_2_, 24 h at 37 °C). Two of them were weighed after 24 h. Then they were dried overnight at room temperature and weighed again. The remaining two gels were stored in 15 mL/20 mmol/L of HEPES buffer with a pH of 7.5 and containing 140 mmol/L of NaCl at 37 °C, for one day after their production. Afterwards, these washed gels were extracted, weighed, dried overnight, and then weighed again. To calculate the exact masses of the gels, the additional masses of all used salts (HEPES, CaCl_2_, NaCl) were subtracted from the result.

### 4.6. Scanning Electron Microscopy (SEM) and Image Analysis

Scanning electron microscopy was performed with a ZEISS “Neon 40“. Pictures of the samples were obtained by using a SE2-detector (for topography) and an InLens detector (for material contrast) at an acceleration voltage of 2 kV.

Fibrinogen hydrogels were prepared as described earlier. A 12 × 12 mm glass slide was used to extract some of the material. Then the sample was dried overnight at room temperature and ambient pressure. Prior to microscopy, all samples were sputtered with a 3 nm thick layer of Au/Pd alloy.

Fiber diameters were determined using GIMP. In each image, five individual fibers were chosen. Their diameter (in pixels) was measured and converted into an actual length.

### 4.7. Rheology

To obtain the first insights into the rheological properties, an Anton Paar MCR 302 was used. All samples were measured with a cone/plate setup. First, the linear viscoelastic regime was identified by means of an amplitude scan. Based on these results, a consecutive frequency sweep was performed at 1% shear deformation. G’ and G’’ were measured from 0.01 Hz–15 Hz. For each measurement, a fresh sample was used.

Pseudo-fibrin hydrogels were prepared as described above (20 mg/mL of protein, 20 mmol/L of HEPES with a pH of 7.5, 100 mmol/L of NaCl, 50 mmol/L of CaCl_2_, 24 h at 37 °C). Fibrin references were prepared analogously but without CaCl_2_. Instead, clotting was induced by adding 5 U/mL of thrombin. In addition, a reference sample containing only fibrinogen was prepared analogously to the pseudo-fibrin samples but without the addition of any trigger salt or enzyme. All samples were prepared twice to have an undamaged one for the measurements.

## Figures and Tables

**Table 1:** Comparison of the initial pseudo-fibrin gels and the washed one in terms of yield and water content.

	Initial Gel	Washed Gel
Fibrinogen content [mg]	17.5 ± 0.1	10.2 ± 0.7
Water content [mg]	637.1 ± 23.4	471.3 ± 10.2
Water content [mg H_2_O/mg protein]	36.4 ± 1.4	46.2 ± 3.3

## Data Availability

Data are contained within the article or Supplementary Materials.

## Supplementary Materials

The following supporting information can be downloaded at: https://www.mdpi.com/article/10.3390/gels9030175/s1, Figure S1: SDS-PAGE analysis of the supplied fibrinogen and thrombin to rule out potential enzyme residuals. No additional enzymes were identified in the supplied fibrinogen (“F”, right). As a reference, thrombin (“T”, left) shows the expected line at 40 kDa; Figure S2: SEM images of Ca^2+^-induced pseudo-fibrin prepared at alkaline pH (10.0–7.5). Up to a pH of 9.5, no qualitative differences in fiber morphology or yield could be determined. At a pH of 10.0 however, the fibers become less defined and coalesce; Figure S3: SEM images of Ca^2+^-induced pseudo-fibrin prepared at a neutral and acidic pH (7.0–5.0). Already at a pH of 6.5, a significant decrease in the fiber yield can be observed. Below this pH, gelation and fibrillogenesis are inhibited; Figure S4: SEM images of fibrin prepared under the same conditions as Ca^2+^-induced hydrogels. Due to the high fibrinogen concentrations combined with the necessary drying step, many fibers coalesced to a dense clot; Table S1: Average diameter of pseudo-fibrin fibers in the pH range from 10.0–6.5. Below a pH of 6.5, no fibers are obtained; Table S2: Average diameter of pseudo-fibrin fibers dependent on the Ca^2+^ concentration. At 50 mmol/L of Ca^2+^, no fibers are obtained; Video S1: Impression of the Ca^2+^-induced hydrogel (in real time).
