# Supplementary material for: Fibrillogenesis and Hydrogel Formation from Fibrinogen Induced by Calcium Salts
Source: Gels. 2023 Feb 22;9(3):175. doi: 10.3390/gels9030175 (PMC10048482; doi:10.3390/gels9030175)
Supplement: Supplementary file 1 [file gels-09-00175-s001.zip › gels-2211229-supplementary.pdf]

Supporting Information

Fibrillogenesis and Hydrogel Formation from Fibrinogen Induced by Calcium Salts

Dominik Hense and Oliver I. Strube *

Institute for Chemical Engineering, University of Innsbruck, Innrain 52c, 6020 Innsbruck, Austria
* Correspondence: oliver.strube@uibk.ac.at; Tel.: +43-512-507-55300

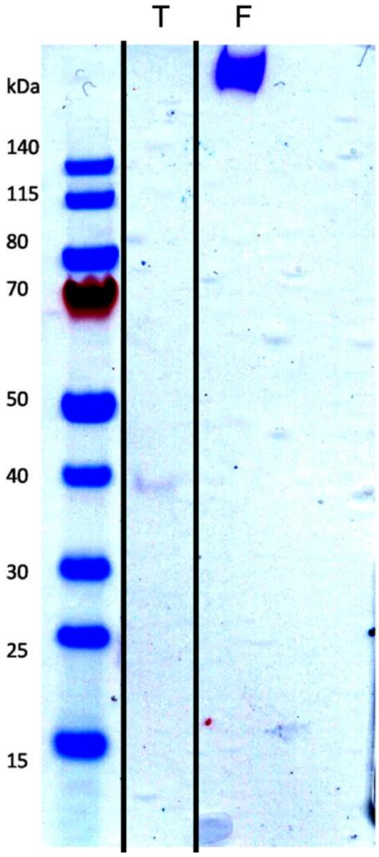

Figure S1. SDS-PAGE analysis of the supplied fibrinogen and thrombin to rule out potential enzyme residuals. No additional enzymes were identified in the supplied fibrinogen ("F", right). As a reference, thrombin ("T", left) shows the expected line at 40 kDa.

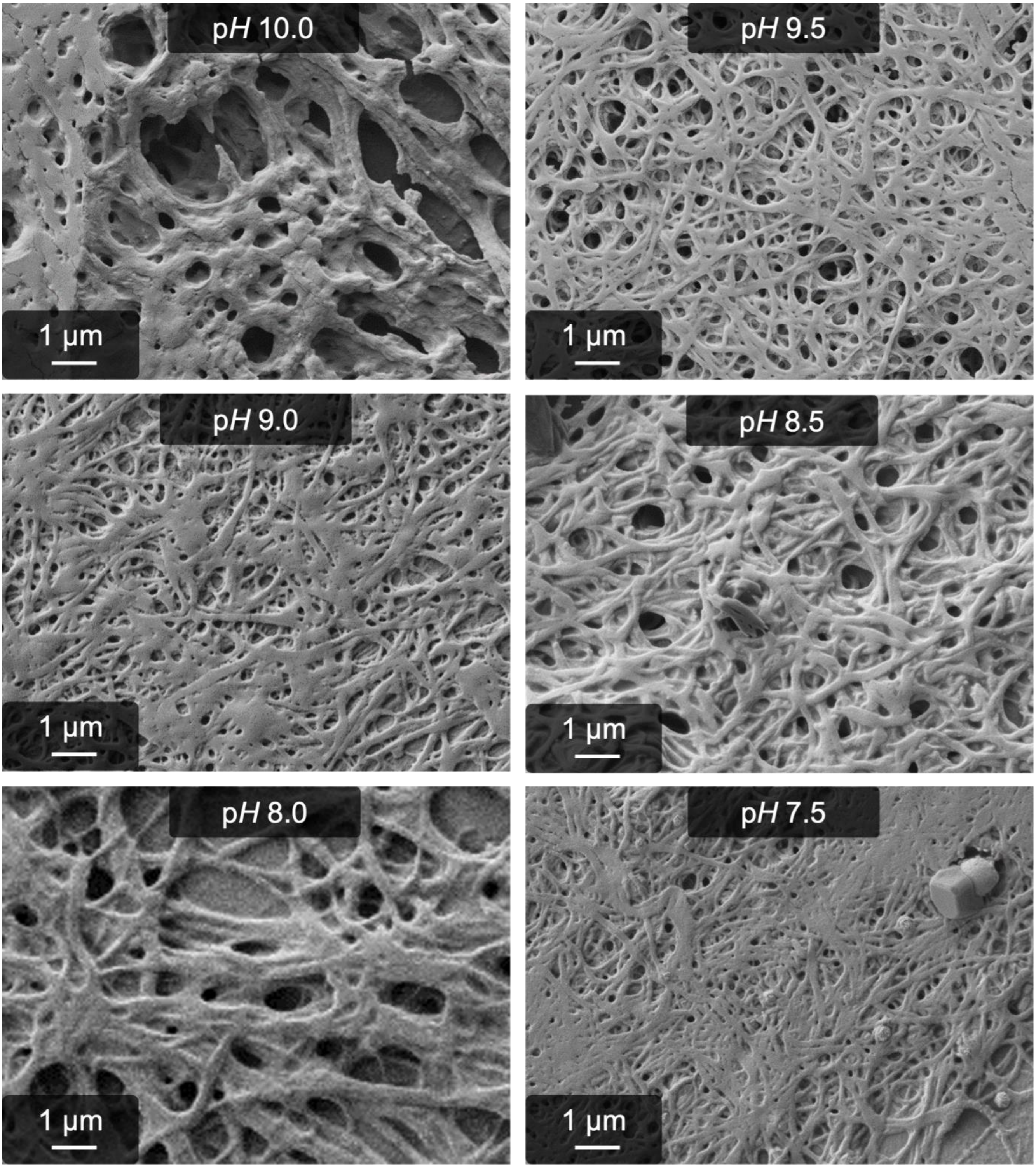

Figure S2. SEM images of Ca^{2+}-induced pseudo-fibrin prepared at alkaline pH (10.0–7.5). Up to pH 9.5, no qualitative differences in fiber morphology or yield could be determined. At pH 10.0 however, the fibers become less defined and coalesce.

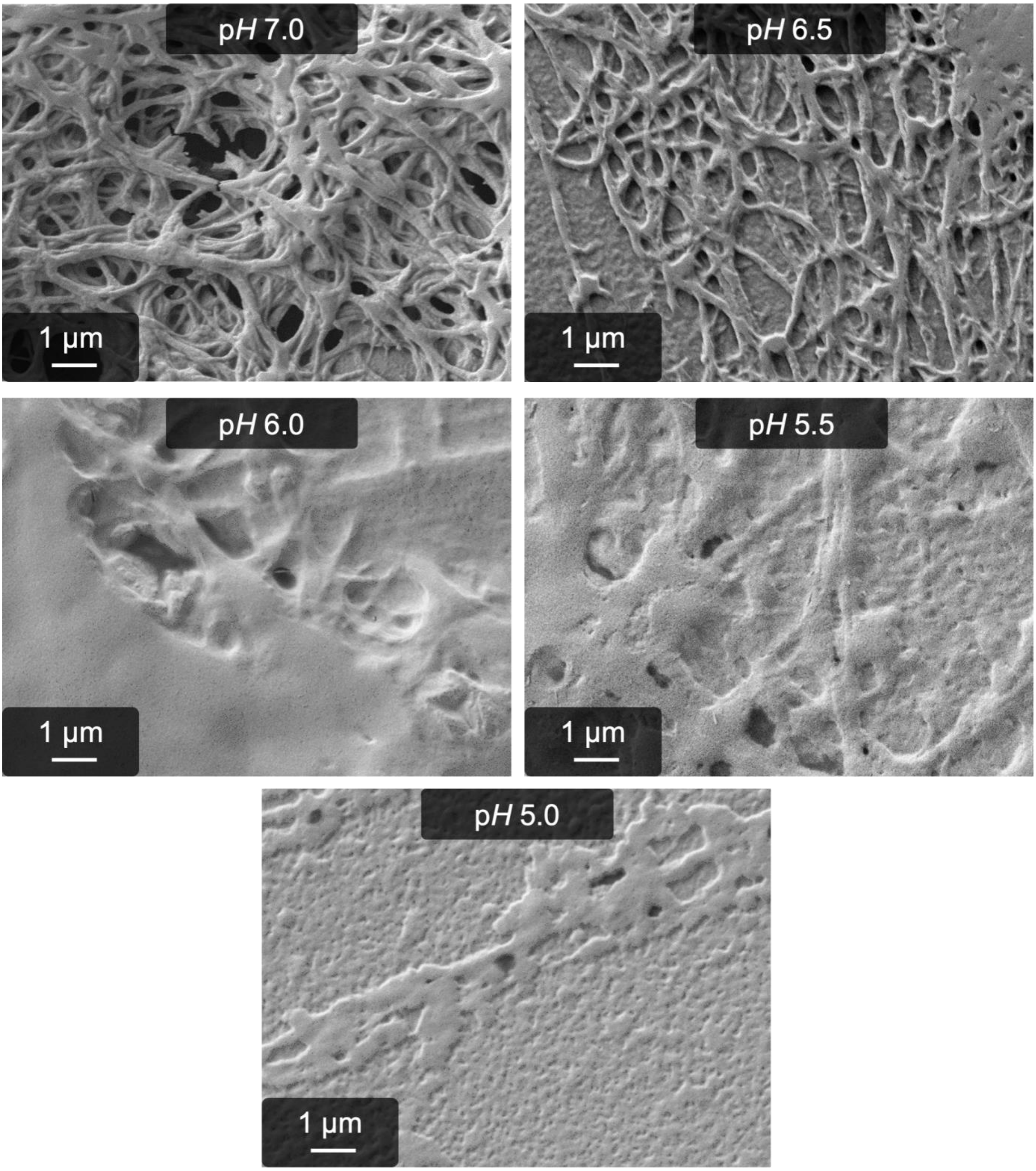

Figure S3. SEM images of Ca^{2+}-induced pseudo-fibrin prepared at neutral and acidic pH (7.0–5.0). Already at pH 6.5, a significant decrease in fiber yield can be observed. Below this pH, gelation and fibrillogenesis are inhibited.

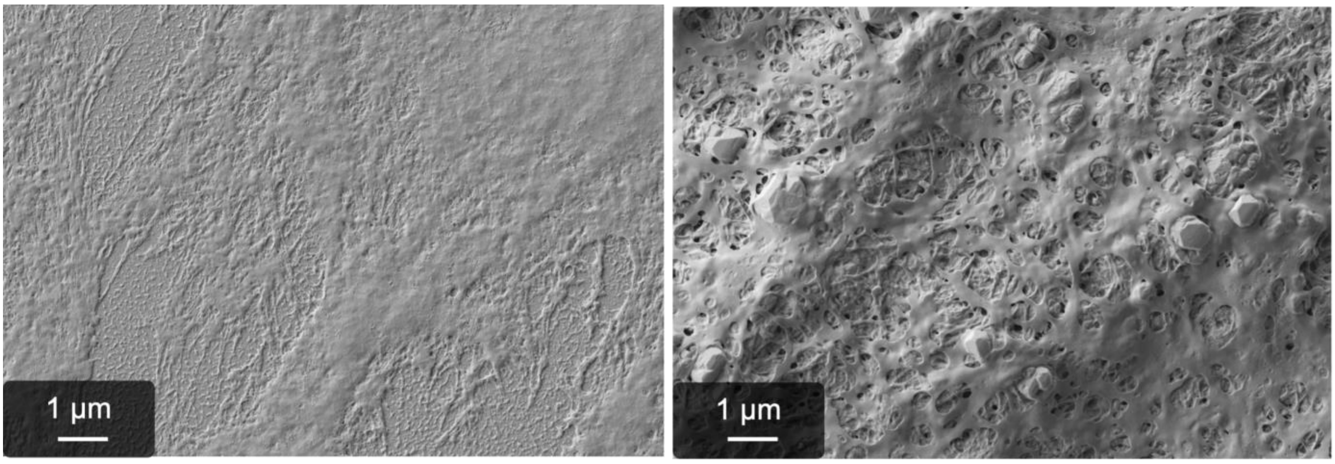

Figure S4. SEM images of fibrin prepared under the same conditions as Ca^{2+}-induced hydrogels. Due to the high fibrinogen concentrations combined with the necessary drying step, many fibers coalesced to a dense clot.

Table S1. Average diameter of pseudo-fibrin fibers in the pH range from 10.0–6.5. Below pH 6.5, no fibers are obtained.

**p*H***	**10.0**	**9.5**	**9.0**	**8.5**	**8.0**	**7.5**	**7.0**	**6.5**
Fiber diameter [nm]	225 ± 145	123 ± 18	144 ± 23	177 ± 43	183 ± 25	125 ± 9	231 ± 20	210 ± 61

Table S2. Average diameter of pseudo-fibrin fibers dependent on the Ca^{2+} concentration. At 50 mmol/L Ca^{2+}, no fibers are obtained.

**c [mmol/L]**	**0.5**	**1**	**5**	**15**	**30**
Fiber diameter [nm]	148 ± 48	131 ± 40	104 ± 9	231 ± 20	174 ± 41

Video S1. Impression of the Ca^{2+}-induced hydrogel (real time).
